# Supplementary material for: Alcohol Consumption Norms and the Favored Alcohol Consumption Policies of Citizens of Seoul
Source: Int J Environ Res Public Health. 2024 Jun 26;21(7):834. doi: 10.3390/ijerph21070834 (PMC11276656; doi:10.3390/ijerph21070834)
Supplement: Supplementary file 1 [file ijerph-21-00834-s001.zip › ijerph-2996205-supplementary.pdf]

# Alcohol Consumption Norms and the Favored Alcohol Consumption Policies of Citizens of Seoul

Table S1. Drinking norms scores according to sociodemographic characteristics and health behaviors

(N=1,001)

| Characteristics | | Drinking norms[1] mean(SE) | | | | | | | | |
|---|---|---|---|---|---|---|---|---|---|---|
| | | Total | ① | ② | ③ | ④ | ⑤ | ⑥ | ⑦ | ⑧ |
| Total | | 2.27(.02) | 2.07(.03) | 2.61(.03) | 2.59(.04) | 1.61(.03) | 3.22(.03) | 2.14(.03) | 2.54(.03) | 1.40(.03) |
| Gender | Female | 2.17(.02) | 1.99(.04) | 2.56(.05) | 2.43(.05) | 1.48(.03) | 3.15(.04) | 1.97(.04) | 2.48(.04) | 1.33(.03) |
| | Male | 2.38(.03) | 2.15(.05) | 2.66(.05) | 2.76(.05) | 1.74(.04) | 3.30(.05) | 2.32(.05) | 2.60(.05) | 1.47(.04) |
| Age(yrs) | 20~39 | 2.45(.05) | 2.28(.06) | 2.93(.06) | 2.93(.06) | 1.71(.05) | 3.44(.05) | 2.14(.05) | 2.73(.06) | 1.47(.05) |
| | 40~59 | 2.22(.05) | 1.99(.05) | 2.53(.05) | 2.47(.05) | 1.60(.04) | 3.19(.05) | 2.13(.05) | 2.49(.05) | 1.38(.04) |
| | ≧60 | 2.09(.05) | 1.89(.06) | 2.25(.06) | 2.30(.07) | 1.48(.05) | 2.95(.06) | 2.15(.06) | 2.35(.06) | 1.32(.05) |
| Marital status | Unmarried | 2.39(.03) | 2.15(.06) | 2.84(.06) | 2.85(.06) | 1.69(.05) | 3.41(.05) | 2.11(.05) | 2.65(.06) | 1.40(.04) |
| | Married | 2.18(.03) | 2.01(.04) | 2.47(.04) | 2.43(.05) | 1.53(.03) | 3.11(.04) | 2.14(.04) | 2.45(.04) | 1.35(.03) |
| | Others[2] | 2.33(.09) | 2.11(.13) | 2.41(.12) | 2.40(.13) | 1.86(.13) | 3.06(.12) | 2.37(.13) | 2.59(.13) | 1.84(.14) |
| Educational status | ≦high school | 2.22(.05) | 2.04(.07) | 2.40(.07) | 2.40(.07) | 1.70(.07) | 3.07(.07) | 2.17(.07) | 2.46(.07) | 1.53(.06) |
| | ≧college,university | 2.29(.02) | 2.08(.04) | 2.68(.04) | 2.66(.04) | 1.58(.03) | 3.27(.04) | 2.13(.04) | 2.57(.04) | 1.36(.03) |
| Job status | having job | 2.33(.03) | 2.15(.04) | 2.67(.04) | 2.67(.04) | 1.66(.03) | 3.26(.04) | 2.18(.04) | 2.59(.04) | 1.45(.03) |
| | No job | 2.11(.04) | 1.85(.06) | 2.44(.06) | 2.37(.07) | 1.45(.05) | 3.11(.06) | 2.03(.05) | 2.41(.06) | 1.26(.04) |
| Monthly househlod income(won) | <2,000,000 | 2.20(.06) | 1.94(.09) | 2.44(.09) | 2.36(.09) | 1.65(.08) | 3.16(.09) | 2.15(.09) | 2.48(.09) | 1.46(.07) |
| | ≦2,000,000<3,000,000 | 2.34(.05) | 2.07(.07) | 2.71(.08) | 2.68(.08) | 1.72(.07) | 3.23(.07) | 2.24(.07) | 2.50(.07) | 1.55(.07) |
| | ≧3,000,000 | 2.27(.03) | 2.10(.04) | 2.61(.04) | 2.62(.04) | 1.57(.03) | 3.23(.04) | 2.11(.04) | 2.57(.04) | 1.33(.03) |
| Number of households | Living alone | 2.28(.06) | 1.94(.08) | 2.63(.09) | 2.69(.09) | 1.65(.08) | 3.27(.09) | 2.11(.08) | 2.58(.09) | 1.40(.07) |
| | ≧2 | 2.27(.02) | 2.09(.04) | 2.60(.04) | 2.58(.04) | 1.60(.03) | 3.21(.03) | 2.14(.03) | 2.53(.04) | 1.40(.03) |
| Smoking | Non-smoker | 2.19(.02) | 1.98(.04) | 2.52(.04) | 2.49(.04) | 1.51(.03) | 3.16(.04) | 2.08(.03) | 2.48(.04) | 1.34(.03) |

| | | | | | | | | | | |
|---|---|---|---|---|---|---|---|---|---|---|
| status | Current smoker | 2.57(.05) | 2.40(.08) | 2.95(.07) | 3.00(.08) | 1.99(.08) | 3.45(.06) | 2.39(.08) | 2.78(.08) | 1.62(.07) |
| Annual drinker | No | 1.86(.05) | 1.61(.08) | 1.98(.08) | 1.97(.08) | 1.34(.06) | 2.79(.08) | 1.94(.08) | 2.09(.09) | 1.19(.04) |
| | Yes | 2.33(.02) | 2.14(.04) | 2.74(.04) | 2.71(.04) | 1.60(.03) | 3.34(.04) | 2.15(.03) | 2.62(.04) | 1.35(.03) |
| High risk drinker | No | 2.24(.02) | 2.03(.04) | 2.59(.04) | 2.56(.04) | 1.56(.03) | 3.23(.03) | 2.09(.03) | 2.53(.04) | 1.32(.03) |
| | Yes | 2.54(.09) | 2.44(.14) | 3.10(.13) | 3.06(.13) | 1.63(.11) | 3.60(.13) | 2.48(.13) | 2.69(.16) | 1.35(.09) |

[1]① It is okay to drink in a park or a mountain reserve ② It is okay to drink alcohol in the daytime ③ It is okay to get a little tipsy ④ It is okay for high school students to drink ⑤ It is okay to drink alone ⑥ It is discourteous to refuse a drink offered by someone else ⑦ It is okay to drink at a convenience store(under internal or external parasols) ⑧ It is okay to lower the punishments for crimes committed after drinking alcohol.

[2]divorce, separated, widowed

Table S2. Scores of preferred alcohol control policies according to sociodemographic characteristics and health behaviors

(N=1,001)

| Characteristics | | Alcohol control policy[1] mean(SE) | | | | | | | | | | | |
|---|---|---|---|---|---|---|---|---|---|---|---|---|---|
| | | Total | ① | ② | ③ | ④ | ⑤ | ⑥ | ⑦ | ⑧ | ⑨ | ⑩ | ⑪ |
| Total | | 2.81(.02) | 2.34(.03) | 2.44(.03) | 2.88(.03) | 2.63(.03) | 3.16(.03) | 3.17(.03) | 2.99(.03) | 2.70(.03) | 3.03(.03) | 2.94(.03) | 2.97(.03) |
| Gender | Female | 2.91(.03) | 2.44(.04) | 2.52(.04) | 2.96(.04) | 2.76(.04) | 3.24(.03) | 3.27(.03) | 3.12(.04) | 2.86(.04) | 3.17(.04) | 3.04(.04) | 3.08(.04) |
| | Male | 2.70(.03) | 2.24(.04) | 2.37(.04) | 2.80(.04) | 2.50(.04) | 3.07(.04) | 3.06(.04) | 2.84(.04) | 2.61(.04) | 2.88(.04) | 2.83(.04) | 2.84(.04) |
| Age(yrs) | 20~39 | 2.68(.03) | 2.27(.05) | 2.33(.05) | 2.77(.04) | 2.44(.05) | 3.00(.04) | 3.00(.05) | 2.92(.05) | 2.65(.05) | 2.91(.05) | 2.80(.05) | 2.83(.05) |
| | 40~59 | 2.87(.03) | 2.37(.04) | 2.49(.04) | 2.91(.04) | 2.71(.05) | 3.22(.04) | 3.27(.04) | 3.07(.04) | 2.80(.04) | 3.09(.04) | 3.02(.04) | 3.03(.04) |
| | ≧60[c] | 2.90(.04) | 2.41(.06) | 2.55(.05) | 3.00(.05) | 2.79(.05) | 3.30(.04) | 3.27(.05) | 2.96(.05) | 2.78(.05) | 3.11(.05) | 3.03(.05) | 3.07(.05) |
| Marital status | Unmarried | 2.71(.03) | 2.28(.05) | 2.36(.05) | 2.77(.04) | 2.49(.05) | 3.07(.04) | 3.06(.05) | 2.95(.05) | 2.66(.05) | 2.95(.05) | 2.81(.05) | 2.83(.05) |
| | Married | 2.87(.02) | 2.37(.04) | 2.48(.04) | 2.97(.03) | 2.74(.04) | 3.22(.03) | 3.26(.03) | 3.03(.04) | 2.78(.04) | 3.08(.03) | 3.04(.04) | 3.05(.04) |
| | Others[2] | 2.83(.07) | 2.51(.12) | 2.63(.10) | 2.84(.11) | 2.59(.13) | 3.11(.10) | 3.10(.09) | 2.83(.10) | 2.90(.09) | 3.06(.11) | 2.86(.11) | 3.02(.10) |
| Educational status | ≦high school | 2.88(.04) | 2.51(.06) | 2.62(.06) | 2.93(.05) | 2.72(.06) | 3.21(.05) | 3.18(.06) | 2.98(.06) | 2.81(.06) | 3.05(.05) | 3.02(.05) | 3.03(.05) |
| | ≧college, university | 2.78(.02) | 2.29(.03) | 2.39(.03) | 2.87(.03) | 2.60(.03) | 3.14(.03) | 3.17(.03) | 2.99(.03) | 2.72(.03) | 3.02(.05) | 2.91(.03) | 2.94(.03) |
| Job status | having job | 2.79(.02) | 2.35(.03) | 2.43(.03) | 2.85(.03) | 2.60(.03) | 3.12(.03) | 3.13(.03) | 2.96(.03) | 2.74(.03) | 3.04(.03) | 2.92(.03) | 2.94(.03) |
| | No job | 2.85(.04) | 2.33(.06) | 2.47(.05) | 2.96(.05) | 2.72(.06) | 3.25(.04) | 3.28(.05) | 3.06(.05) | 2.73(.06) | 3.01(.05) | 2.99(.05) | 3.02(.05) |
| Monthly househlod income (won) | <2,000,000 | 2.83(.05) | 2.38(.08) | 2.47(.08) | 2.85(.07) | 2.67(.08) | 3.19(.07) | 3.17(.07) | 2.92(.07) | 2.80(.08) | 3.07(.07) | 2.95(.08) | 3.03(.07) |
| | ≦2,000,000 <3,000,000 | 2.76(.04) | 2.30(.06) | 2.40(.06) | 2.79(.06) | 2.55(.07) | 3.09(.06) | 3.11(.06) | 2.94(.06) | 2.74(.06) | 2.97(.06) | 2.96(.08) | 2.94(.06) |
| | ≧3,000,000 | 2.82(.02) | 2.35(.04) | 2.45(.03) | 2.92(.03) | 2.65(.04) | 3.17(.03) | 3.19(.03) | 3.02(.03) | 2.73(.03) | 3.04(.03) | 2.93(.03) | 2.96(.03) |
| Number of households | Living alone | 2.74(.05) | 2.17(.07) | 2.31(.07) | 2.85(.07) | 2.49(.08) | 3.18(.07) | 3.11(.08) | 3.02(.08) | 2.75(.08) | 2.97(.07) | 2.90(.08) | 2.88(.08) |
| | ≧2 | 2.82(.02) | 2.37(.03) | 2.47(.03) | 2.89(.03) | 2.65(.03) | 3.16(.03) | 3.18(.03) | 2.98(.03) | 2.74(.03) | 3.04(.03) | 2.94(.03) | 2.98(.03) |
| Smoking status | Non-smoker | 2.86(.02) | 2.39(.03) | 2.47(.03) | 2.94(.03) | 2.68(.03) | 3.22(.03) | 3.22(.03) | 3.07(.03) | 2.80(.03) | 3.09(.03) | 2.99(.03) | 3.03(.03) |
| | Current smoker | 2.60(.04) | 2.16(.07) | 2.36(.06) | 2.67(.06) | 2.44(.07) | 2.91(.06) | 2.97(.06) | 2.67(.06) | 2.51(.06) | 2.79(.06) | 2.74(.06) | 2.71(.06) |

| | | | | | | | | | | | | | |
|---|---|---|---|---|---|---|---|---|---|---|---|---|---|
| Annual drinker | No | 3.18(.05) | 2.85(.07) | 2.91(.07) | 3.26(.06) | 3.07(.07) | 3.48(.05) | 3.46(.05) | 3.25(.07) | 3.18(.06) | 3.30(.07) | 3.25(.07) | 3.28(.06) |
| | Yes | 2.72(.02) | 2.18(.03) | 2.32(.03) | 2.79(.03) | 2.52(.03) | 3.11(.03) | 3.13(.03) | 2.93(.03) | 2.63(.03) | 2.98(.03) | 2.88(.03) | 2.89(.03) |
| High risk drinker | No | 2.81(.02) | 2.32(.03) | 2.43(.03) | 2.88(.03) | 2.63(.03) | 3.18(.03) | 3.19(.03) | 3.00(.03) | 2.74(.03) | 3.04(.03) | 2.95(.03) | 2.96(.03) |
| | Yes | 2.52(.09) | 1.82(.11) | 2.05(.11) | 2.69(.12) | 2.34(.12) | 2.94(.13) | 3.00(.13) | 2.73(.12) | 2.34(.13) | 2.79(.12) | 2.74(.12) | 2.82(.12) |

[1]① Liquor price increase ② Restrictions on liquor promotion activities ③ Restriction on places that sell alcoholic beverage ④Restriction on liquor selling times ⑤ Expansion of drinking prohibitions in public places ⑥ Restrictions on drinking at rallies(events) ⑦ Attach warning images to bottles ⑧ Restrictions on the provision of free alcoholic beverages at local events ⑨ Restrictions on liquor advertisements ⑩ Restrictions on drinking scenes in visual media ⑪ Restrictions on liquor advertisements in visual media [2]divorce, separated, widowed

Table S3. Correlations between drinking norms items and preferences for alcohol control policies

(N=1,001)

| Variables | | Drinking norms | | | | | | | | Alcohol control policy | | | | | | | | | | |
|---|---|---|---|---|---|---|---|---|---|---|---|---|---|---|---|---|---|---|---|---|
| | | ① | ② | ③ | ④ | ⑤ | ⑥ | ⑦ | ⑧ | ① | ② | ③ | ④ | ⑤ | ⑥ | ⑦ | ⑧ | ⑨ | ⑩ | ⑪ |
| Drinking norms[1] | ① | 1 | | | | | | | | | | | | | | | | | | |
| | ② | .525** | 1 | | | | | | | | | | | | | | | | | |
| | ③ | .406** | .608** | 1 | | | | | | | | | | | | | | | | |
| | ④ | .426** | .327** | .367** | 1 | | | | | | | | | | | | | | | |
| | ⑤ | .235** | .525** | .466** | .159** | 1 | | | | | | | | | | | | | | |
| | ⑥ | .391** | .233** | .303** | .370** | .137** | 1 | | | | | | | | | | | | | |
| | ⑦ | .454** | .430** | .403** | .277** | .384** | .333** | 1 | | | | | | | | | | | | |
| | ⑧ | .388** | .200** | .260** | .641** | .024 | .467** | .277** | 1 | | | | | | | | | | | |
| Alcohol control policy[2] | ① | −.160** | −.298** | −.246** | −.039 | -.254** | −.081* | −.191** | .063* | 1 | | | | | | | | | | |
| | ② | −.153** | −.282** | −.259** | −.052 | -.263** | −.089** | −.232** | .017 | .666** | 1 | | | | | | | | | |
| | ③ | −.313** | −.273** | −.249** | -.177** | -.186** | −.185** | −.251** | -.160** | .476** | .460** | 1 | | | | | | | | |
| | ④ | −.232** | −.313** | −.292** | -.116** | -.245** | −.111** | −.239** | −.039 | .559** | .521** | .597** | 1 | | | | | | | |
| | ⑤ | −.393** | −.297** | −.286** | -.245** | -.139** | −.287** | −.283** | -.278** | .310** | .323** | .545** | .449** | 1 | | | | | | |
| | ⑥ | −.323** | −.261** | −.278** | -.232** | -.138** | −.235** | −.290** | -.236** | .261** | .298** | .480** | .390** | .614** | 1 | | | | | |
| | ⑦ | −.251** | −.235** | −.251** | -.185** | -.148** | −.247** | −.214** | -.169** | .408** | .372** | .474** | .451** | .508** | .488** | 1 | | | | |
| | ⑧ | −.237** | −.261** | −.238** | -.084** | -.209** | −.144** | −.277** | −.039 | .474** | .477** | .490** | .548** | .426** | .435** | .511** | 1 | | | |
| | ⑨ | −.223** | −.240** | −.249** | -.218** | -.168** | −.193** | −.241** | -.197** | .333** | .317** | .449** | .421** | .453** | .437** | .505** | .539** | 1 | | |
| | ⑩ | −.209** | −.259** | −.262** | -.135** | -.195** | −.139** | −.237** | -.122** | .340** | .389** | .434** | .476** | .465** | .453** | .461** | .539** | .654** | 1 | |
| | ⑪ | −.227** | −.262** | −.277** | -.160** | -.214** | −.150** | −.271** | -.125** | .370** | .375** | .469** | .470** | .480** | .470** | .510** | .551** | .700** | .804** | 1 |

**p<.001, *p<.05 bold values indicate negative correlation.

[1]① It is okay to drink in a park or a mountain reserve ② It is okay to drink alcohol in the daytime ③ It is okay to get a little tipsy ④ It is okay for high school students to drink ⑤ It is okay to drink alone ⑥ It is discourteous to refuse a drink offered by someone else ⑦ It is okay to drink

at a convenience store(under internal or external parasols) ⑧ It is okay to lower the punishments for crimes committed after drinking alcohol.

[2]① Liquor price increase ② Restrictions on liquor promotion activities ③ Restriction on places that sell alcoholic beverage ④Restriction on liquor selling times ⑤ Expansion of drinking prohibitions in public places ⑥ Restrictions on drinking at rallies(events) ⑦ Attach warning images to bottles ⑧ Restrictions on the provision of free alcoholic beverages at local events ⑨ Restrictions on liquor advertisements ⑩ Restrictions on drinking scenes in visual media ⑪ Restrictions on liquor advertisements in visual media
